# A feasibility randomised controlled trial of Empowered Conversations: training family carers to enhance their relationships and communication with people living with dementia

**DOI:** 10.1186/s40814-026-01799-6

**Published:** 2026-04-02

**Authors:** Lydia Morris, Cassie Eastham, Chris Sutton, Yeliz Prior, Yvonne Sylvestre, Gemma Shields, John Keady, Cathy Riley, Mal Walters, Warren Mansell

**Affiliations:** 1 Division of Psychology and Mental Health, School of Health Sciences, Clinical Psychology Doctorate Team, University of Manchester, Manchester, M13 9PL UK; 2 Manchester Mental Health NHS Foundation Trust, Manchester, UK; 3 Division of Population Health, Health Services Research & Primary Care, University of Manchester, Manchester, UK; 4 School of Health and Society, University of Salford, Salford, UK; 5 Division of Nursing, Midwifery and Social Work, University of Manchester, Manchester, UK; 6 Open Doors Research Group, Greater Manchester Mental Health NHS Foundation Trust, Salford, UK; 7 Curtin School of Population Health Curtin University, Perth, Australia

**Keywords:** Dementia, Communication, Caregivers, Psychosocial intervention, Psychoeducational intervention, Multicomponent intervention

## Abstract

**Background:**

The primary objective of this UK-based trial was to investigate the feasibility of conducting a multi-centre randomised controlled evaluation trial of Empowered Conversations (EC). EC is a 6-session group psychosocial intervention for informal (family) care partners of people living with dementia. The two key feasibility objectives were to establish whether recruitment levels and retention to follow-up were sufficient for a multi-centre evaluation trial to be feasible. Secondary objectives were as follows: to estimate potential effectiveness on a range of candidate primary outcome measures and their standard deviations; to identify the most appropriate primary outcome measure for a multi-centre evaluation trial; to obtain additional evidence regarding proof of concept; to establish the optimum way of evaluating cost-effectiveness in the evaluation trial.

**Methods:**

The feasibility trial used a pragmatic data-collector blind parallel two-group RCT design with two arms (EC intervention plus treatment as usual, and treatment as usual waitlist control). There was a 2:1 allocation in favour of the EC arm. Participants completed baseline outcome measures including measures of their psychological health, quality of life and service use. These were repeated after 6 months.

**Results:**

Seventy-five care partners were recruited. The average number of people randomised per month was 8.9, consistent with the pre-specified average recruitment rate of 6 to 10 carers per month sufficient for proceeding to a multi-centre trial. A total of 58 (77%) participants were retained at 6 months follow-up meeting the amber stop-go criterion (65%–<80%; green ≥ 80% retention).

**Conclusion:**

The trial indicated the feasibility of progressing to an evaluation trial of EC. Recruitment was at a sufficient level for a multi-centre trial across three proposed sites. Retention to follow-up was close to the green criterion, and ways of increasing retention in the evaluation trial have been identified.

**Trial Registration:**

ISRCTN15261686; Registered 02/03/2022 https://www.isrctn.com/ISRCTN15261686

**Supplementary Information:**

The online version contains supplementary material available at 10.1186/s40814-026-01799-6.

## Key messages regarding feasibility

1. The trial was conducted primarily to examine the feasibility of recruitment pathways and retention to the Empowered Conversations intervention. Other key feasibility objectives included identifying the most appropriate primary outcome measure.
2. The trial recruited to time and to target. The Perceived Stress Scale was identified to be the best primary outcome option.
3. The main implication was that retention to follow-up was slightly lower than expected and so some changes have been made to the proposed multi-centre trial to improve this.

## Background

The personal, social and economic impact of dementia is substantial. Family and informal carers provide intense practical support, whilst coming to terms with changes in their relationship [1]. Within the United Kingdom (UK) alone it is estimated that 944,000 people have dementia [2] and there are approximately 700,000 informal carers [3]. Worldwide there were over 55 million people living with dementia in 2020; it is predicted that this number will almost double every 20 years, reaching 78 million in 2030 and 139 million in 2050 [4].

Informal care, defined as unpaid support given to a family member, partner or friend who could not otherwise cope because of their physical or mental health needs [5], is an essential component of dementia care. Based on costings for 2015 in the UK, unpaid dementia care incurs a cost of £13.9bn per year, compared with £15.7bn spent on formal health and social care [6]. [Hereafter ‘care partner’ will be used for informal/family carers].

About 40% of care partners of people living with dementia experience significant depression or anxiety [7]. As care partners witness significant changes within someone to whom they are very close, they can experience consequent changes in role and significant losses. For example, a spouse or child may feel increasingly cast in a parental role and care partners commonly express that they can feel they have ‘lost’ the person to the ongoing changes of dementia [8]. Care partners can experience major relationship changes and complex grief, in addition to practical stressors [1, 8]. Furthermore, a systematic review and meta-ethnographic synthesis of 25 high-quality studies found that the two main reasons for behaviours of people living with dementia being reported as challenging by carers were firstly, changes in communication and relationships and, secondly, perceived transgressions against social norms associated with ‘misunderstandings about behaviour’ in the relative with dementia, such as attributing challenging behaviour to the person rather than seeing it as a symptom of dementia [9]. These findings indicate the importance of understanding of dementia (psychoeducation) and skills to manage changes in communication.

Given the high social, economic and individual costs of dementia, and the impact on care partners, it is important to ensure evidence-based treatments are available. Sixty-four percent of care partners in England said they had limited support for the range of psychological and social needs they experienced [10]. Post-diagnostic support for UK care partners remains limited [11, 12].

In addition to the limited availability of psychosocial interventions to support care partners within the UK [12], there has been limited evaluation of the interventions that are available. Across recent reviews of psychosocial interventions for informal carers of people living with dementia, including meta-reviews [13–15], key recommendations focused on the need to improve the quality of the research evidence, to focus on contextual and implementation mechanisms, and to evaluate the needs of different carer subgroups [14, 16].

Our team have developed the Empowered Conversations (EC) (6-session group, delivered weekly) manualised theory-informed psychosocial intervention in collaboration with care partners and people living with dementia [17–20]. EC is based on the Communication Empowerment Framework (CEF) which integrates evidence-based models (of psychological health, communication and relationships) to address the specific psychological, relationship and communication needs of care partners [1]. It is a psychosocial intervention, with a focus on communication, relationships and stress; it is integrative and draws on a range of theoretical accounts, such as Perceptual Control Theory [21] and Attachment Theory (a key theory of how we relate to each other in times of stress), given its unique focus on relationship stress. The CEF proposes that the psychological challenges of being a carer for a person living with dementia can be addressed through exploring new perspectives on their relationships that allow both parties to regain and maintain control over valued aspects of the conversation, and over enduring values within their lives. There are three main pathways through which EC is hypothesised to reduce carer distress (e.g. reduce stress), and improve wellbeing [1]. Two of these mechanisms are improving relationships and enhancing communication. The third is goal conflict or ambivalence. Therefore, measures of these are included to explore proof-of-concept and inform the future trial.

EC is delivered in a group format and includes multiple components, including both educational and therapeutic components. The most recent UK NICE dementia guidance recommends carer interventions are delivered in groups [22]. EC has been delivered and evaluated online and in person, via an initial pre-post, follow-up (uncontrolled) feasibility study of the in-person version and two qualitative studies of informal carers’ experience of in-person and online EC [17–20]. The pre-post-follow-up study of in-person EC (*N* = 159) demonstrated that carer stress was significantly reduced, and communication significantly improved over time following participation in the course [17]. Twenty-eight of the carers were interviewed after EC and twenty-seven described feeling able to better connect with the person they support after attending EC [18]. During the COVID-19 pandemic, an online (video-conference, e.g. Zoom platform) version of EC was developed and was evaluated in this feasibility trial. Both in-person and online versions of EC are manualised and intervention components delivered are almost identical. The minor differences are to allow successful delivery via these two different media; for example, a group exercise that involves splitting attendees into two groups in person is delivered as a whole group exercise online. The nested qualitative study, which involved interviews with 15 carers regarding online EC is reported elsewhere [20].

This feasibility RCT, with nested qualitative study, was conducted to determine the feasibility of progressing to a multi-centre trial and inform the design of a multi-centre RCT to evaluate the effectiveness of EC. The primary feasibility outcomes were levels of recruitment and retention to follow-up. An important secondary feasibility outcome was cost-measurement feasibility. The feasibility of using the Clinical Dementia Rating Scale (CDR) to measure dementia severity was also examined; however, the primary feasibility criteria (and stop–go criteria) was based on recruitment levels and retention to follow-up.

## Methods

### Trial objectives

The primary aim of the study was to establish the feasibility of examining the clinical and cost-effectiveness of EC within a multi-centre RCT.

The key objectives were:

1. To establish recruitment pathways.
2. To identify facilitators and barriers to recruitment.
3. To estimate retention levels and response rates to questionnaires.
4. To obtain additional evidence regarding proof of concept.
5. To estimate potential effectiveness on a range of candidate primary outcome measures and their standard deviations (SDs).
6. To identify the most appropriate primary outcome measure for a multi-centre evaluation trial.
7. To establish the optimum way of evaluating cost-effectiveness in a multi-centre evaluation trial.

A secondary feasibility objective was to examine the feasibility of measuring severity of dementia symptoms via the CDR within this context.

### Trial design

The trial used a pragmatic data-collector blind parallel two-group RCT design. The two arms were the EC intervention (plus Treatment as Usual) and Treatment as Usual (TAU) waitlist control. There was a 2:1 allocation in favour of the EC intervention arm. Unbalanced randomisation was chosen to provide more information on aspects of EC, such as barriers to participation and intervention acceptability. Baseline and 6-month follow-up data were collected. Participants in the TAU arm were offered EC after completing their 6-month follow-up questionnaires.

The trial also included a nested qualitative study [20].

Trial registration: ISRCTN15261686 (registered on February 3rd, 2022); protocol version 2.6 21/11/2023. The trial protocol has been published [23].

No major changes were made to the trial protocol following the commencement of recruitment, but a minor procedural change was made. It was anticipated that a proportion of care partners would opt for in-person assessments, but all participants opted to complete assessments online. So, in response to this, additional text reminders and an additional phone call were used to maximise assessment completion in the absence of a scheduled appointment with a researcher.

### Participants, interventions, and outcomes

#### Study setting

The study took place across the ten boroughs (Bolton, Bury, Manchester, Oldham, Rochdale, Salford, Stockport, Tameside, Trafford, and Wigan) within Greater Manchester, a metropolitan county in the Northwest of England with varying levels of deprivation. Indices of multiple deprivation are based on several factors and include income and employment deprivation (low income and low employment rates); they also include health (risk of premature death and impairment of life quality through poor mental/physical health) and education (skills and attainments). Some Greater Manchester boroughs, specifically Manchester and Salford, are amongst the most deprived in England [23]. The study was hosted by Greater Manchester Mental Health NHS Foundation Trust with a study site at Pennine Care NHS Foundation Trust, and we recruited through both trusts.

Recruitment was also through the third sector and community organisations across Greater Manchester, and Join Dementia Research. Members of the study team attended groups and meetings, both online and in person, to talk about participation in the trial and raise awareness; for example, the Research Associate for the trial (CE) attended carer groups and Memory and Assessment Team meetings across the ten boroughs. The study was also promoted on social media channels (Twitter and Facebook), organisational newsletters/blogs, and via professional and service user/carer networks. Care partners either self-referred to the project or were referred by healthcare professionals and service providers.

### Ethical approval and consent to participate

All procedures contributing to this work comply with the ethical standards of the relevant national and informed consent was obtained for all participants. All procedures were approved by the Wales Research Ethics Committee in 2022 (REC: 22/WA/0010).

All participants gave informed consent. In line with the Mental Capacity Act, the research team assumed capacity of the person living with dementia unless it was established otherwise. All practicable steps were taken to help the person make the decision to take part in the study. Information and consent resources were provided in an easy-to-read format, explanations presented in various ways, and it was ensured that the time and location of the appointment optimised the person’s participation.

The researcher referred to the study’s standard operating procedure for assessing capacity. If the researcher perceived that the person living with dementia lacked capacity to consent to participate, this was documented and discussed with the person and the carer. The researcher checked whether the carer was suitable and willing to act as a personal consultee. If they are not suitable or willing, another friend/relative will be sought to act as a personal consultee.

The researcher would then discuss the person’s participation with the personal consultee. Following the capacity assessment and best interest forms, the researcher sought the consultee’s views on whether it was in the person’s best interest to take part in the research. This decision was documented.

### Eligibility criteria

People must:

- be the current unpaid or informal carer for someone living with dementia (any sub-type or severity);
- live in Greater Manchester;
- be aged 18 or over;
- have capacity to give informed consent for the study;
- have sufficient English language skills to understand and participate in the training and research activities;
- be interested in taking part in a training course for carers of people living with dementia.

Where both care partner and person living with dementia consented, the CDR was used to assess the person living with dementia’s level of cognitive impairment. The care partner’s eligibility to participate was not conditional on the person living with dementia being offered or completing the CDR. This aspect of the study was to establish the feasibility of measuring severity of those living with dementia’s level of cognitive impairment in this manner and we did not want this to be a barrier to care partner participation.

### Patient and Public Involvement and Engagement (PPIE)

PPIE work was conducted with members of the Salford Open Doors research group and two designated public advisors. The group met three times a year. Public advisors also attended the quarterly trial steering committee meeting and bimonthly trial management group meetings. Tasks undertaken by the PPIE representatives included: monitoring study progress; and providing care partner perspectives on aspects of the trial including outcome measures, recruitment, and dissemination.

### EC intervention

EC is a six-session group-based intervention; each session lasts 2 h and is held at the same time and day for the duration of the course. It is a multi-component intervention in that it includes psychoeducation, psychotherapeutic and support group elements. For the trial, it was delivered online using Zoom by two trained facilitators from Age UK Salford; eight facilitators delivered EC in total during the trial. Facilitators followed a course manual to deliver a structured framework of core topics, discussions, and activities over 6 weeks. Please see Supplementary material for a description of core intervention components. However, the facilitators also had flexibility to adapt the material to the different needs of participants, including optional and extra activities that can be used if appropriate.

#### EC facilitators

EC facilitators are generally care partners, or former care partners, of people living with dementia. They come from a range of professional backgrounds, including medical and community sectors. 

#### Intervention fidelity

 Facilitators were trained to deliver the course using the manual and had attended an EC course before their training began. New facilitators received 9 h of one-to-one training provided across the duration of two EC courses. New facilitators began to deliver aspects of these courses with support from an experienced facilitator, weekly debriefs, and supervision. All course facilitators accessed weekly supervision with an experienced facilitator and monthly external clinical supervision; existing facilitators had already been trained using the same training approach.

Fidelity and competence of the course facilitators was monitored using an adapted version of the checklist that has been used in two previous studies of a group-based intervention using similar techniques to EC (the Take Control Course) [24, 25]. Facilitator fidelity was other-rated by someone familiar with the course but not directly involved in the research (e.g. the Clinical project director), by rating for two of the sessions in which each facilitator was observed whilst delivering different sessions on different courses during the RCT.

### Treatment as usual (TAU)

TAU was the medical, psychological, and social support that is available to the care dyad within their local area. This included, but was not limited to, services such as NHS memory assessment or community mental health teams, dementia cafés, social care, and carers support groups. There was no restriction on TAU. This was a pragmatic trial and preventing care partners from accessing services would have been unethical.

### Feasibility criteria and outcome measures

The study measured outcomes in terms of the feasibility of conducting a multi-centre study, which focused on whether participant recruitment and retention to follow-up were sufficient for an evaluation trial to be feasible. Feasibility criteria were measured in terms of:

Recruitment numbers per month—If an average of 6–10 carers were recruited per month from the proposed Greater Manchester recruitment site, then this would be expected to be at a sufficient level for a multi-centre trial. This would need to be fulfilled in addition to the below stop/go criterion regarding retention.

### Retention rate recorded as the number of randomised participants who remain in the study at the 6-month follow-up. Stop/go criteria for retention were:

Green (progress to full trial): At least 80% retention:

Amber (full trial considered feasible if reasons for poor retention identified and can be addressed): 65%– <80% retention.

Red (unlikely to progress to full trial): Below 65% retention.

A secondary objective was to identify the most appropriate primary clinical outcome measures. Participants completed ten clinical outcome measures at baseline and at 6-month follow-up to address objectives 4–6. Potential primary outcome measures were: Short Sense of Competence Questionnaire, Dyadic Relationship Scale (Caregiver), Carer Communication Questionnaire, Perceived Stress Scale, Hospital Anxiety & Depression Scale (HADS). In addition, C-DEMQOL (carer version Dementia QOL measure), Caregiving Ambivalence Scale, Bristol Activities of Daily Living Scale (BADLS). Objective 7 related to cost-effectiveness and the outcomes used were EQ-5D-5L and health and social care service use [23]. Participants were also asked to record key demographic data at baseline and complete a feedback form when they finished the course sessions.

The CDR scale was used as a measure to directly assess the supported person’s level of cognitive impairment. This clinical interview assessment was conducted at baseline with the care dyad. The feasibility of using this in the proposed multi-centre trial was established via levels of uptake.

The feasibility of proceeding to a multi-centre study (objectives 1, 2 and 3) was judged on the recruitment and retention of participants in relation to pre-determined stop/go criteria [23].

### Sample size

The target sample size for the RCT was 75 randomised (50 EC:25 TAU). This is a typical sample size for feasibility trials and, assuming a minimum of 80% retention (60 participants), will enable the SD to be estimated with satisfactory precision [26] and the overall retention rate to be estimated by a 95% confidence interval with width 19.2%. It will also enable estimation of efficacy (Standardised Effect Size [SES]) using an 80% confidence interval with width ≤ 0.4. However, to allow for pre-randomisation withdrawals and to ensure each course cohort was an appropriate size (6–10 participants), the recruitment target was up to 90.

### Assignment of interventions

#### Sequence generation

A computer-generated randomisation list was generated by the independent randomisation service Sealed Envelope, using random permuted blocks of selected block sizes to allocate participants in a 2:1 ratio, in favour of the EC intervention arm.

Randomisation was performed at the individual level. However, after consent had been provided and baseline assessments taken, individual randomisations were delayed until there were sufficient participants to start a course; at this stage, a set of individual randomisations was performed and a course was formed from those who were allocated to the EC intervention arm.

### Allocation concealment mechanism and method of implementation of the allocation sequence

On completion of the consent form and baseline measures, the researcher (CE) sent the participant’s details to the EC administrator, who then randomised the participant to the treatment or control group using the online Sealed Envelope application, thus ensuring allocation concealment.

### Blinding

The trial was data-collector blind. We could not blind participants nor those delivering the EC intervention. Emergency unblinding could have occurred if the facilitators or researchers identified a high risk of self-harm or suicide, or of harm from others (e.g. safeguarding concerns); in this scenario it would be likely that unblinding would be needed to best support the participant’s well-being.

The protocol stated that the research team remained blinded until after the participant completed the 6-month follow-up questionnaires. This was followed, but in practice, given that all participants self-completed outcome measures online, any potential impact of unblinding was considerably reduced. The statistical team remained blinded until the Statistical Analysis Plan was approved.

### Statistical analysis

To describe feasibility of recruitment, retention, and study participants’ characteristics, we used appropriate descriptive statistics. Summary measures were presented as mean and standard deviations for continuous (approximately) symmetrically distributed variables, and frequencies and percentages for categorical. Pooled standard deviations (SD) were also presented for outcome measures. Overall retention rates and completion rates for individual outcome questionnaires were estimated using point estimates with 95% binomial CIs.

Analyses to assess proof of concept and proof of efficacy were by ‘intention-to-treat (ITT)’ as CONSORT guidelines recommend to fully preserve the benefit of randomisation. This analysis approach requires that participants be retained and analysed in the allocated treatment group. No imputation of missing outcome data was performed; however, for missing baseline values of the corresponding outcome data, we used simple mean imputation (across the groups) to avoid exclusion of such participants in the complete-case analysis. We assumed all missing outcome data was missing at random (MAR) conditional on any variable included in the analysis model, and so independent of the values of the unobserved data themselves. Analyses of clinical efficacy outcomes are likelihood-based and therefore consistent with the MAR assumption.

Mixed-effects regression analysis was used to analyse the candidate primary outcome measures (at follow-up), and for the three pathways targeted by EC (ambivalence [goal conflict], relational stress and communication). In each case, models include the treatment factor (fixed effect), the baseline value of the corresponding outcome measure (fixed effect) and ‘course’ (random effect in a partially nested model).

Potential proof of concept was examined using adjusted point estimates and confidence intervals (CIs), ranging from 75 to 95% confidence (steps of 5%, following the approach proposed by Lee et al., 2014), for the between-group differences in means for the candidate primary outcomes measures obtained from the analyses described above (Lee et al., 2014). This approach is based on a minimally important difference (MID) between trial arms and is therefore more appropriate than formal hypothesis testing when a study is underpowered. We explored the perceived size of MID during this study. A clinically meaningful difference between arms for a simple and low-intensity intervention such as EC is generally around an effect size of 0.3 [27].

Exploratory analysis was conducted to inform a cost-effectiveness analysis within a definitive trial, including: an analysis of the range of services used and ability of participants to report complete service use data; the ability of utilities (informed by the EQ-5D-5L) to discriminate between groups based on changes in clinical outcomes; factors likely to influence the incremental cost per QALY ratio.

Full details of the quantitative analyses were included in a Statistical and Health Economic Analysis Plan (SHEAP). The approved version of the SHEAP is available on the University of Manchester repository (Figshare).

### Harms

The primary participants in this trial were community-based informal carers caring for people living with dementia. Although this is a group that does not have a particular elevated risk for Adverse Event (AE) or Serious Adverse Event (SAE), we followed the host NHS organisation’s guidance for recording and reporting adverse events for non-CTIMPs.

## Results

### Participant characteristics

Participants in the EC and TAU groups were relatively similar in most demographic and baseline characteristics as shown in Table 1. Those allocated to the EC intervention arm were 4 years older on average than participants allocated to the TAU group. The proportion of participants caring for their spouse was moderately higher in the EC group, whilst the proportion of participants caring for their parents was higher in the TAU group.

**Table 1:** Demographic and baseline characteristics of trial participants by treatment allocated

		**EC intervention**	**TAU**	**Total**
		***n*** = 51	***n*** = 24	***n*** = 75
**Demographics**				
Age (years)	Mean(SD)	63.2 (9.5)	59.0 (10.6)	61.8 (10.0)
	Range	46.0 to 82.0	42.0 to 83.0	42.0 to 83.0
	Missing	2		2
Gender	Male	12 (24)	2 (8)	14 (19)
	Female	37 (73)	22 (92)	59 (79)
	Missing	2 (4)	0 (0)	2 (3)
Ethnicity	White	46 (90)	23 (96)	69 (92)
	Mixed/Multiple ethnic groups	1 (2)	0 (0)	1 (1)
	Asian/Asian British	0 (0)	1 (4)	1 (1)
	Black/African/Caribbean/Black British	1 (2)	0 (0)	1 (1)
	Other ethnic group	1 (2)	0 (0)	1 (1)
	Missing	2 (4)	0 (0)	2 (3)
Sexual identity	Straight/heterosexual	49 (96)	23 (96)	72 (96)
	Prefer not to say	0 (0)	1 (4)	1 (1)
	Missing	2 (4)	0 (0)	2 (3)
**Caring role**				
Who do you care for	Spouse	26 (51)	10 (42)	36 (48)
	Long-term partner	2 (4)	0 (0)	2 (3)
	Parent	18 (35)	12 (50)	30 (40)
	Sibling	1 (2)	2 (8)	3 (4)
	Friend	1 (2)	0 (0)	1 (1)
	Other relationship	1 (2)	0 (0)	1 (1)
	Missing	2 (4)	0 (0)	2 (3)
Dementia diagnosis	Alzheimer’s	23 (45)	9 (38)	32 (43)
	Vascular	8 (16)	6 (25)	14 (19)
	Parkinson’s	1 (2)	0 (0)	1 (1)
	Frontotemporal	0 (0)	1 (4)	1 (1)
	Lewy bodies	1 (2)	2 (8)	3 (4)
	Mixed	12 (24)	5 (21)	17 (23)
	Other type of dementia	4 (8)	1 (4)	5 (7)
	Missing	2 (4)	0 (0)	2 (3)
Primary carer	Yes	46 (90)	17 (71)	63 (84)
	Missing	2 (4)	0 (0)	2 (3)
**Residence**				
Do you live with the person you care for?	Yes	33 (65)	13 (54)	46 (61)
	Missing	2 (4)	0 (0)	2 (3)
**Family and dependents**				
Other living in your household	1	29 (57)	15 (63)	44 (59)
	2	10 (20)	3 (13)	13 (17)
	3	4 (8)	2 (8)	6 (8)
	4 or more	4 (8)	2 (8)	6 (8)
	Not applicable	2 (4)	2 (8)	4 (5)
	Missing	2 (4)	0 (0)	2 (3)
People living in your household*	Spouse	41	18	59
	One child < 18	3	5	8
	2 or more children < 18	3	0	3
	Other family members	14	4	18
	Other dependents	2	4	6
	Share rent & space	3	0	3
Household income for the year (pounds)	Less than 15,000	2 (4)	2 (8)	4 (5)
	15,000—29,999	12 (24)	5 (21)	17 (23)
	30,000—44,999	11 (22)	4 (17)	15 (20)
	45,000—59,999	6 (12)	2 (8)	8 (11)
	60,000—74,999	1 (2)	2 (8)	3 (4)
	75,000—89,999	4 (8)	0 (0)	4 (5)
	125,000 and above	1 (2)	0 (0)	1 (1)
	Prefer not to say	10 (20)	8 (33)	18 (24)
	Missing	4 (8)	1 (4)	5 (7)
Highest degree earned/level of school completed	Secondary education	8 (16)	5 (21)	13 (17)
	Further education e.g. A-level, C&G, foundation degree, GNVQ/NVQ up to level 3,	12 (24)	7 (29)	19 (25)
	Higher education e.g. Undergraduate degree, HNC, HND	14 (27)	8 (33)	22 (29)
	Postgraduate education e.g. Masters, Doctorate, Postgraduate Diploma, PGCE, prof	14 (27)	2 (8)	16 (21)
	Prefer not to say	1 (2)	1 (4)	2 (3)
	Missing	2 (4)	1 (4)	3 (4)
Currently employed*	Employed for salary	18	10	28
	Employed on a volunteer/non-salaried basis	2	0	2
	Self-employed	2	2	4
	Out of work and looking for work	1	2	3
	Retired	27	8	35
	Unable to work	2	2	4
	Other	0	1	1
Changed due to Coronavirus pandemic	Yes	3 (6)	4 (17)	7 (9)
	Missing	2 (4)	0 (0)	2 (3)
Hours of work if in paid employment	Work fewer than 30 h a week	6 (12)	5 (21)	11 (15)
	Work between 30 and 40 h a week	13 (25)	4 (17)	17 (23)
	Work between 40 and 50 h a week	0 (0)	2 (8)	2 (3)
	Missing	32 (62)	13 (57)	45 (60)
Main job (SOC)	1. Managers, directors & senior officials	7 (14)	3 (13)	10 (13)
	2. Professional occupations	19 (37)	8 (33)	27 (36)
	3. Associate professional occupations	9 (18)	3 (13)	12 (16)
	4. Administrative & secretarial occupations	6 (12)	7 (29)	13 (17)
	5. Skilled trades occupations	1 (2)	0 (0)	1 (1)
	6. Caring, leisure and other service occupations	2 (4)	1 (4)	3 (4)
	7. Sales & customer service occupations	1 (2)	0 (0)	1 (1)
	8. Process, plant & machine operatives	1 (2)	0 (0)	1 (1)
	9. Elementary occupations	1 (2)	0 (0)	1 (1)
	Missing	4 (8)	2 (8)	6 (8)
English as second language	Yes	0 (0)	1 (4)	1 (1)
	Missing	3 (6)	0 (0)	3 (4)

*There can be more than one answer per carer

There were also moderate observed differences in the financial assistance received, being higher in the TAU group. The proportion of participants with a postgraduate education was higher in the EC group.

### EC courses and facilitator fidelity

Seven EC courses were delivered as part of the trial. Six was the mean number of participants per course and the mean number who provided outcome data was five.

A total of 12 sessions for 6 facilitators were rated, in which each facilitator was observed whilst delivering different sessions on different courses during the RCT.

Fidelity and competence were generally high (see Table 2). However, facilitators were less skilled in managing time effectively than they were in other aspects, with this being rated as met 67% of the time.

**Table 2:** Tabulation of the individual items on the fidelity and competence checklist

1. Did the facilitator cover the key features of the course manual?	*n* (%)
Yes	12 (100%)
2. Did the facilitator use the manual resources?	
Yes	12 (100%)
3. Did the session appear to be well structured?	
Yes	12 (100%)
4. Did the facilitator manage the time effectively, i.e. allow sufficient time for each section and finish on time?	
Yes	8 (67%)
Some	4 (33%)
5. Did the facilitator encourage carers to move towards a curious position when considering what might be going on for the person living with dementia?	
Yes	10 (83%)
Some	2 (17%)
6. Did the facilitator effectively demonstrate the use of the course tools e.g. Fried Egg, Upward Arrow, Control Continuum etc.?	
Yes	11 (92%)
Some	1 (8%)
7. Did the facilitator provide reflective space for carers to share how the previous weeks learning had been processed?	
Yes	12 (100%)
8. Did the facilitator adapt to carers needs flexibility within the sessions and course?	
Yes	10 (83%)
Some	2 (17%)

#### Harms

There were no AEs or SAEs reported during the trial.

### *Recruitment and retention (see CONSORT, *Fig. 1*)*

**Fig. 1:**
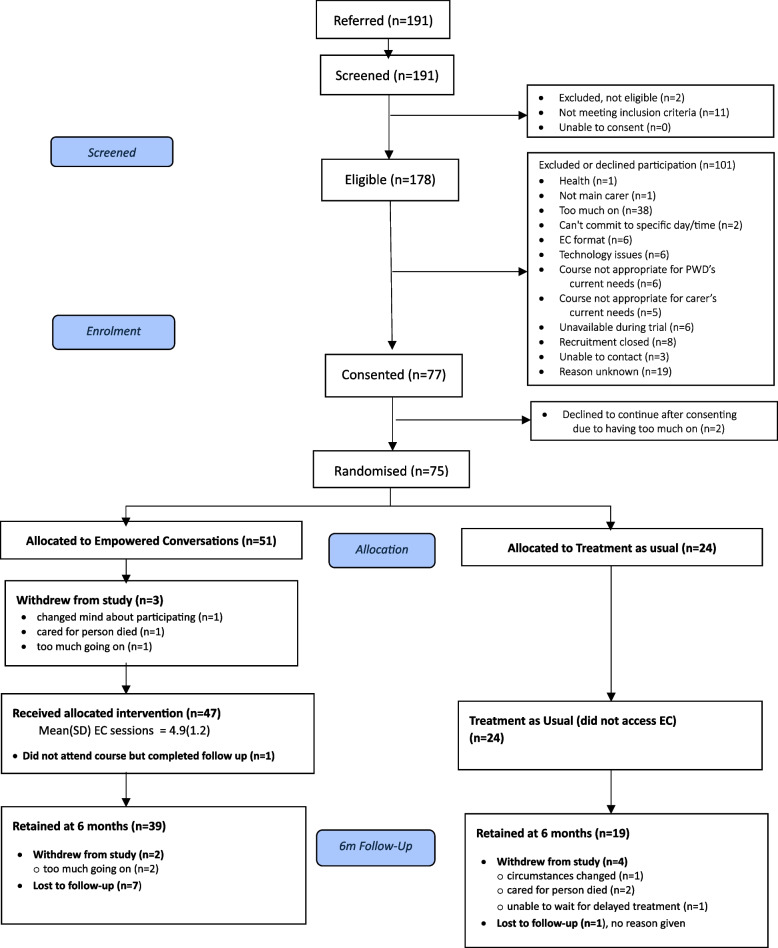
CONSORT flowchart

Objectives 1 and 2 focused on establishing recruitment pathways and identifying facilitators/barriers to recruitment.

There was a total of 191 carers referred and screened for eligibility of whom 178/191 (93%) were deemed eligible. Of the eligible carers, 103/178 (58%) declined and a total of 75/178 (42%) were randomised. A total of 51/75 (68%) of the carers were allocated to the EC intervention arm and 24/75 (32%) to TAU. A detailed breakdown of reasons why participants declined or were not eligible is included in the CONSORT diagram (Fig. 1). The most common reason for not participating in the trial was ‘having too much on’ (*n* = 38). Other reasons for not participating included: ‘issues with EC format’ (*n* = 6) and ‘technology’ (*n* = 6). Qualitative data (reported in detail in 20) also indicated that the online delivery mode could be a barrier and was not every carer’s preference.

#### Recruitment numbers per month

Participant recruitment began on 1 st March 2022 and the final participant was randomised on 11th November 2023. The overall average monthly rate randomised was 75/8.4 = 8.9 participants per month, and therefore the pre-specified average recruitment rate of 6 to 10 carers per month from the Greater Manchester recruitment site for proceeding to a multi-centre trial was met.

A total of 17/75 (23%) carers agreed to complete the Clinical Dementia Rating (CDR) scale with the person living with dementia they were supporting. Only five carers of the 58 carers who declined provided a reason for this and the reasons included that the person living with dementia did not acknowledge their diagnosis, they believed the person living with dementia would not be able to participate, the person living with dementia lived outside Greater Manchester.

Objective 3 was concerned with the estimation of retention levels, response rates to questionnaires and the stop–go criteria regarding retention. Of the 75 participants randomised, 9 (12%) withdrew from the study and 3 (4%) withdrew from the EC intervention. An additional 8 (11%) participants were lost to follow up, three of whom were those who withdrew from the EC intervention and, despite agreeing to remain in follow-up, did not provide outcome data. Where available, reasons for withdrawals are shown in the CONSORT flowchart (Fig. 1).

#### Stop–go criteria

*Participant retention* was defined a priori as ‘a participant completing some or all of the 6-month outcome questionnaires’. A total of 58 (77%) participants were retained at 6-months follow-up. The Amber criterion for retention was met; 77%, 95% CI (66% to 86%) carers were retained at 6 months. 80% or higher was the Green criteria.

*Retention in terms of the EC intervention* was defined (in advance) as a carer attending three or more out of the six EC sessions. The CONSORT flowchart shows that 47/51 (92%) of the carers allocated to the EC intervention arm commenced the intervention and attended 4.9 (SD = 1.9) sessions on average. A total of 44/51 (86%) carers attended at least 3 sessions of the EC therapy, and from the remaining 3 carers, 2/3 attended 2 EC sessions, and 1/3 attended 1 session only. Thus, 86% (95% CI 74% to 94%) carers were deemed adherent to the EC intervention. The average number of sessions attended for the adherent carers was 5.1 (SD = 0.9).

### Questionnaire completion rates

In evaluating questionnaire completion, we firstly evaluated item non-response within individual questionnaires (health measurement tools). Item response was high with at least 92% (69/75) of the carers completing all items at baseline and 91% (53/58) at 6 months for all the questionnaires, with the exception of the C-DEMQOL where item response was generally lower. The proportion of carers completing all items for the C-DEMQOL subscales ranged from 53% (40/75) to 95% (71/75).

In the absence of tool-specific guidance on handling item non-response, we imputed missing item scores as the mean of the scores for the completed items prior to computation of the scale or subscale scores if no more than 25% of the items were missing for a particular scale or subscale. Otherwise, the scale or subscale score was deemed to be missing.

The completion rates (baseline to 6 months) for all the questionnaires except the C-DEMQOL were consistent with the participant overall retention rate at 6 months; 77%, 95% CI (66% to 86%).

### Proof of concept (Objective 4)

Table 3 shows the treatment effects at 6-months for the proof-of-concept outcomes. Between-group differences are the proposed mediators for the efficacy outcomes, with 95% confidence intervals.

**Table 3:** Treatment effects at 6 months for the ‘proof-of-concept’ outcomes, results generated from Mixed-effect models

	Scale range	***N***	**Effect estimate**	**95% CI**	**… in favour of …**
Carer Communication Questionnaire (CCQ)	7 to 56	58	4.24	(1.42 to 7.06)	EC
Caregiving Ambivalence Scale (CAS)	0 to 18	58	0.42	(−0.81 to 1.64)	TAU

### Estimate potential effectiveness on a range of candidate primary outcome measures, and their standard deviations (Objective 5)

Table 4 shows the treatment effects at 6-months for the candidate proof-of-efficacy outcomes with a range of confidence intervals, and the corresponding SES (with standardised 95% confidence interval), where standardisation was performed using the pooled within-group standard deviation. Most treatment effect estimates are small with 95% CIs including zero. The exception was the Carer Communication scale that showed a statistically significant effect, with a large SES of 0.83 = 4.24/5.12 (standardised 95% CI 0.28 to 1.38). EC led to a small reduction in stress on the PSS compared to control, for which the SES estimate was 0.13 (95% CI −0.32 to 0.57).

**Table 4:** Treatment effects at 6 months for clinical or health outcomes: results generated from mixed-effect models

	**Scale range**	***N***	**Effect estimate**	**… in favour of …**	**95% CI**	**90% CI**	**85% CI**	**80% CI**	**75% CI**	**SD**_pooled_	**Standardised effect size (standardised 95%CI)**
Short Sense of Competence Questionnaire (SSCQ)*	7 to 35	58	0.15	EC	(−1.94 to 2.25)	(−1.61 to 1.91)	(−1.39 to 1.69)	(−1.22 to 1.52)	(−1.08 to 1.38)	5.26	0.03 (−0.37 to 0.43)
Dyadic relationship scale (DRS)											
Dyadic Strain (DS)*	0 to 15	58	1.13	TAU	(−0.34 to 2.61)	(−0.11 to 2.37)	(0.05 to 2.22)	(0.17 to 2.10)	(0.27 to 2.00)	3.11	0.36 (−0.11 to 0.84)
Positive Dyadic Interaction (DI)*	0 to 18	58	−0.88	TAU	(−2.61 to 0.84)	(−2.33 to 0.56)	(−2.15 to 0.38)	(−2.01 to 0.24)	(−1.89 to 0.13)	3.31	−0.27 (−0.79 to 0.25)
Hospital Anxiety and Depression Scale (HADS)											
HADS Anxiety*	0 to 21	58	1.39	TAU	(−0.31 to 3.09)	(−0.04 to 2.82)	(0.14 to 2.64)	(0.28 to 2.50)	(0.39 to 2.39)	3.73	0.37 (−0.08 to 0.83)
HADS Depression*	0 to 21	58	−0.51	EC	(−1.43 to 0.42)	(−1.28 to 0.27)	(−1.19 to 0.17)	(−1.11 to 0.10)	(−1.05 to 0.03)	1.62	−0.31 (−0.88 to 0.26)
HADS-T score*	0 to 42	58	1.12	TAU	(−1.05 to 3.30)	(−0.70 to 2.95)	(−0.47 to 2.72)	(−0.30 to 2.54)	(−0.15 to 2.40)	3.88	0.29 (−0.27 to 0.85)
Perceived Stress Scale (PSS)*	0 to 40	58	−0.84	EC	(−3.80 to 2.12)	(−3.32 to 1.65)	(−3.01 to 1.33)	(−2.78 to 1.10)	(−2.58 to 0.90)	6.71	−0.13 (−0.57 to 0.32)
C-DEMQOL											
C-DEMQOL: Personal	6 to 30	58	0.32	EC	(−1.57 to 2.20)	-	-	-	-	-	-
C-DEMQOL: Wellbeing	6 to 30	57	0.08	EC	(−1.99 to 2.16)	-	-	-	-	-	-
C-DEMQOL: Carer role	6 to 30	55	1.42	EC	(−0.88 to 3.71)	-	-	-	-	-	-
C-DEMQOL: Feelings	6 to 30	57	1.48	EC	(−0.65 to 3.62)	-	-	-	-	-	-
C-DEMQOL: Carer support	6 to 30	51	−0.07	TAU	(−2.32 to 2.19)						
C-DEMQOL: Total	30 to 150	51	−0.33	TAU	(−11.61 to 10.96)						

*Candidate primary outcomes

Effect estimate = EC intervention + TAU—TAU

Standardised effect size (SES) = Effect estimate/SD_pooled_; the corresponding 95%CI is calculated by dividing the limits of the 95% CI for the effect estimate

### Identify the most appropriate primary outcome measure for a multi-centre evaluation trial (Objective 6)

As reported in Table 2, EC led to a small reduction in stress on the PSS, for which the SES estimate was 0.13 (95% CI −0.32 to 0.57) (unstandardised effect estimate 0.84; SD 6.71).

In parallel to the quantitative data collection and analysis, we collected qualitative data to inform this aim. The qualitative data overall is reported in more detail in Eastham et al., 2024 (*N* = 15 care partners). Most care partners reported more than one outcome as important, but the most commonly reported outcome that was viewed as important to carers was ‘my own mental health’; identified by 9 carers. Several specifically mentioned stress. Feedback from our PPIE group and a key stakeholders and PPIE consultation session (11 informal carers for people with dementia, 10 service providers/clinicians, 3 researchers) also supported this as the key outcome (we conducted a ‘rating of outcomes’ exercise within the consultation session). We therefore use the PSS within the following sample size calculation for an evaluation trial.

To achieve 80% (90%) power to detect a SES of 0.3 (typical for trials of psychological interventions) would require 510 (678) participants (assuming 80% retention [i.e. 20% attrition] in each arm), an EC intervention-group clustering ICC of 0.02, and a course cohort size of 5 (the mean number of participants per group providing outcome data) in the EC arm. For the putative primary outcome, the PSS, the point estimate of the ICC was 0.00 (to 2 d.p.) although precision was poor, due to the number of EC course groups being only 7. For a three-site trial, recruiting over 18 months, a sample size of 510 (required to achieve 80% power) appears to be feasible, although this would require a slightly higher recruitment rate per site than achieved in this trial (9.4 per month, compared with 8.9 per month in this single-site feasibility trial). The required sample size could be reduced due to the correlation between baseline and 6-month outcomes. Walters et al. (2019) have suggested that baseline-outcome correlations are typically in the range 0.4–0.6, which if as large as 0.6, can result in a 36% reduction in the sample size: in the above specification, that would result in a target sample size of 328 rather than 510 (and recruitment rate per site per month for an 18-month recruitment period of around 6.0 rather than 9.4).

### Establishing the optimum way of evaluating cost-effectiveness in an evaluation trial (Objective 7)

The cost-effectiveness component of the feasibility focused on assessing the completeness of existing measures to assess their suitability for use in a full trial. In line with current NICE recommendations, the mapping function developed by the Decision Support Unit (DSU) using the ‘EEPRU dataset’ was used to calculate utility values from the EQ-5D-5L data. Baseline utility was 0.749 (SD 0.229, *n* = 72). As would be expected, this is below EQ-5D-5L population norms for similar age groups (0.810 for ages 55–64). At baseline, the most affected domains were anxiety and depression, and pain and discomfort, with 63.9% and 59.7% of participants reporting some problems with these respectively. Roughly three-quarters (73.3%) of participants had complete EQ-5D-5L domain responses at both time points. Therefore, the EQ-5D-5L was well completed and appears to reflect some of the burden of caring when compared to population norms. Existing evidence supports the use of the EQ-5D for utility in studies related to dementia [28]. However, the EQ-5D’s sensitivity to changes resulting from a communication intervention is unknown, and caution has been noted when applying the EQ-5D in populations with communication challenges [29]. Although the cited study was using EQ-5D with people living with dementia and the current study used it with care partners, a full economic evaluation should consider whether additional measures are needed to supplement cost-effectiveness findings derived from the EQ-5D.

The service-use questionnaire collected participant-reported health and social care use, with a focus on adapting the questionnaire to improve completeness in a full trial. At baseline, 65% of participants answered all key questions about the use of services by category. Some of the comments on the service-use questionnaire raise concerns about whose service-use participants reported (their own, the person they care for, or a combination of the two). The service-use data collection provides information that will help to refine a questionnaire for use in a full trial (e.g. simplifying categories and ensuring text is more specific to guide participants).

## Discussion

The primary aim of the trial was to establish the feasibility of evaluating EC within a well-powered multi-centre RCT. The key feasibility outcomes were: levels of recruitment and retention to follow-up. Regarding the primary progression criteria (recruitment), the average monthly rate randomised was 8.9 care partners per month and therefore the pre-specified average recruitment rate of 6 to 10 carers per month for proceeding to a multi-centre trial was met. Regarding the second primary progression criteria (retention to follow-up), a total of 58 (77%) participants were retained at 6-month follow-up. The retention level was therefore slightly below the 80% ‘Green’ criterion to definitely proceed to an evaluation trial but at the higher end of the ‘Amber’ criterion range of 65%–<80% retention (full trial considered feasible if reasons for poor retention can be addressed). Proposed ways of increasing retention are detailed subsequently. Therefore, progression to an evaluation trial was deemed feasible. The results of the cost-effectiveness component also indicated feasibility of progression; however, it was identified that the service-use questionnaire needed refining somewhat to support participants’ responses.

There have been several large meta-reviews of the effectiveness of carer interventions, but there is still limited consensus regarding which intervention to offer to whom and when [15, 30]. A recent meta-review recommended that the classification of interventions should be more transparent and consistent and that interventions should be developed to meet carers’ changing needs [14]; in order to achieve this, information must be provided on what specific needs the intervention addresses and via what mechanisms [31] (e.g. by providing a detailed conceptual framework, such as the CEF, [1]). There are three main pathways through which EC is hypothesised to reduce carer distress (e.g. reduce stress) and improve wellbeing [1]. Two of these pathways are: improving relationships and enhancing communication. Within this trial, EC had a large effect on communication, with estimated SES of 0.83 (95% CI 0.28 to 1.38) on the Carer Communication scale.

One of the challenges in evaluating and implementing care partner interventions is the range of needs a carer will have and that these will change over time [14]. In addition, some carers would not consider themselves to have mental health needs (even if they would meet diagnostic criteria) due to perceiving their response to be due to the ongoing stress of caring; the availability of time and resources can also be barriers to accessing interventions [32, 33]. One response to the changing needs of carers is using a range of measures in evaluating carer outcomes, which makes it harder to compare results across interventions. However, for interventions like EC that do not require clinically significant levels of need (e.g. a diagnosable mental health problem) and are primarily delivered by community and third sector services, it does not make sense to only use clinical outcome measures. Therefore, we include a range of measures that assess key mechanisms (proof-of-concept measures), as well as clinical health outcomes more commonly used in other carer studies. Although these ‘proof-of-concept’ measures (such as communication) are likely to influence health outcomes (such as stress), the majority of our candidate primary outcomes were established health outcomes. Relational stress, or strain, was included as a candidate primary outcome. We included this to measure stress regarding the relationship between the care partner and the person living with dementia, but this could also be considered a proof-of-concept measure.

Effect sizes for differences between the EC intervention and control group were generally small on both proof-of-concept and candidate proof-of-efficacy measures. Given that this was a feasibility trial it was not powered to detect differences between groups and so confidence intervals are wide. EC led to a small reduction in stress on the PSS, for which the standardised effect size estimate was 0.13 (95% CI −0.32 to 0.57; effect estimate 0.84; SD 6.71). Furthermore, there were some differences between the EC intervention and control group; for example, the proportion of participants caring for their spouse was moderately higher in the EC group, whilst the proportion of participants caring for their parents was higher in the TAU group. These results, in addition to feedback within the qualitative component of the trial and PPIE consultation, indicated that stress was the most appropriate primary outcome for the proposed evaluation trial. In addition, an earlier pre-post-follow-up study of the in-person version of EC (*N* = 159) found a SES on the PSS from pre-treatment to 4-month follow-up of 0.48.

Overall, the trial indicated feasibility of progressing to an evaluation trial. Recruitment was at a sufficient level for a multi-centre trial across three proposed sites (Greater Manchester; Lancashire; London). We propose retaining a 2:1 allocation ratio for this trial as we expect it will be more attractive than a trial with a 1:1 ratio, and only slightly less efficient due, in part, to an expected clustering in the EC intervention arm only (meaning that 1:1 allocation will not be the most efficient design). Assuming an ICC of 0.02 in the EC intervention arm, the target sample size for the proposed trial is 336 (accounting for attrition). For a three-site trial, each site recruiting over 17 months, a recruitment rate of 6.59 participants per site per month would be required (compared with 8.93 participants per month in our feasibility trial). As mentioned previously, retention to follow-up was at the high end of the Amber criteria (77%). Three ways of increasing retention in the evaluation trial have been identified: (1) Use of text reminders and follow-up phone calls, as well as email reminders (a greater number of participants than expected opted to complete baseline and follow-up measures online); (2) Providing vouchers (not offered in the feasibility trial); (3) If it does not seem likely that participants will complete all outcome measures, as a last resort, we will have an option (via phone) to just complete the primary outcome (with other key outcomes, if possible). Fidelity and competence of the facilitators was generally high; however, it was recognised that a more detailed fidelity checklist would provide a clearer indication of any aspects of EC that were not being delivered as planned and would provide more information regarding the training and supervision needs of facilitators. Such a detailed fidelity checklist is in development.

The main limitation of this study is the relatively low representation of minoritised ethnic communities. There are well-documented barriers to carers from minoritised communities accessing dementia research, interventions and NHS services [34, 35]. Whilst the evidence for what facilitates access is less well established for dementia carers, the available evidence indicates that minoritised ethnic groups may not access dementia and mental health services due to factors such as stigma and language barriers [36], and so it is important to offer culturally relevant community-based interventions in relevant languages [37–39]. In addition, detailed qualitative interviews (*N* = 10) were conducted with Punjabi Sikhs regarding the cultural appropriateness of EC and adaptations that would make EC more culturally appropriate [40]. Key findings included the utility of: offering EC in person and in community hubs (unless the stigma of being at a community hub could limit accessibility); running the intervention using facilitators with knowledge of Punjabi Sikh culture; representing more people who look and sound like the attendees. In response to this, a budget for translation and interpreters and for additional targeted resources (to better represent minoritised groups) would be included in the costings for a larger trial. In-person delivery should also be offered within this trial. Furthermore, additional training should be provided to facilitators and facilitators recruited from minoritised groups.

As detailed in the previous two paragraphs, the proposed multi-centre evaluation trial will follow the same 2:1 allocation ratio as the feasibility trial and will be conducted with carers. The intervention will be the same, with some minor enhancements to ensure diverse voices are better represented, such as having specific prompts to ask about cultural heritage and including examples with greater cultural diversity. Based on learning from this feasibility trial, the main differences in the evaluation trial will be: (a) providing vouchers; (b) providing EC in person as well as online; (c) not utilising the CDR because only 23% of carers and people living with dementia completed this. In conclusion, the findings indicate that progression to a full evaluation trial is warranted. As well as the proposed trial testing the effectiveness of EC, it will also allow us to explore whether modality (online or in-person delivery) affects outcomes. The evaluation trial has been funded by the NIHR (NIHR208874).

## Supplementary Information

Additional file 1. Supplementary Material

## Data Availability

The dataset supporting the conclusions of this article is available in the Figshare repository, https://figshare.manchester.ac.uk/articles/dataset/Dataset_Empowered_Conversations_feasibility_trial/28902908?file=54093422
