# Supplementary material for: A feasibility randomised controlled trial of Empowered Conversations: training family carers to enhance their relationships and communication with people living with dementia
Source: Pilot Feasibility Stud. 2026 Apr 2;12:67. doi: 10.1186/s40814-026-01799-6 (PMC13169528; doi:10.1186/s40814-026-01799-6)
Supplement: Supplementary file 1 — Additional file 1. Supplementary Material [file 40814_2026_1799_MOESM1_ESM.docx]

Supplementary material: Key session components within the Empowered Conversations course

| **Week** | **Key session components** | **Description** |
| --- | --- | --- |
| **Week 1 - Introducing Curiosity** | Pause for Breath | This is an opportunity to pause and breathe to facilitate responding in a more helpful way. If stressed or frustrated this affects other people (as well as ourselves) and will spill over into the conversations we have. |
|  | The Kitwood Flower | Kitwood's Flower highlights the essential psychological needs of all individuals. This is shared in a short film with an opportunity for participants to comment. |
|  | What is Dementia? | Key facts about dementia are discussed to ensure that we all have a shared understanding. |
|  | Emotions Part 1 and Part 2 | Activity using post-it notes to enable participants to share how they feel talking to the person living with dementia that they support. They then consider how the person living with dementia might feel. |
| **Week 2 - What gets in the way of our conversations?** | What gets in the way of you having the conversations you want to have? | Discussion and exercise to illustrate some barriers to good conversations |
|  | Complexity - Professor Alison Wray - Dementia: The Communication Disease | Outlines key points regarding communication in the context of supporting someone living with dementia. For example, that communication is already a complex process; the importance of knowing where our knowledge overlaps with other peoples’ knowledge and some of the challenges that this can cause. |
|  | Invitation to Respond | This introduces a technique called an invitation to respond - a way into a conversation that does not start with a question. |
|  | Self-Care Battery | A self-care tool that explores the need to check in with our ‘batteries’ (resources) when planning for the day, consider ways to top up ‘batteries’ and identify what drains ‘batteries’. |
| **Week 3 - Stop, Listen, Look** | Jack & Lillian video | Jack and Lillian are a person living with dementia and their care partner. These videos illustrate some of the complexities and facilitators of dialogue between a particular individual living with dementia and their long-term partner. |
|  | What is Non-Verbal communication? | Interactive exercise and discussion to identify experiential elements of non-verbal communication |
|  | 5-minute Relaxation Technique | Self-Care tool to build on the pause for breath and encourage participants to check in with themselves and their bodies. |
| **Week 4 - Empathy and Memory** | Reflections - in pairs | Building on the listening exercise, offering a space to reflect on the course, material and any changes or challenges they have faced using the material. |
|  | Building on Empathy | This exercise encourages participants to see the individual person not just the dementia by sharing who they are, their passions, their life. |
|  | Types of Memory - Film and discussion | The film highlights that memory has different aspects and that not all types of memory are the same or equally impacted by dementia. This is to help identify strengths, e.g. procedural memory can be easier to access for longer for some individuals with dementia and therefore routine tasks can be easier to do in familiar settings. |
| **Week 5 - Building on Strengths and Resources** | Control Continuum | Using visual scaling to enable individuals to consider how much influence they have in key aspects of their life. |
|  | Upward Arrow | Upward arrow resembles the downward arrow technique used within CBT (apart from the arrows are progressing upwards). But the questions fundamentally differ, and the end point is clarifying the goals and values that are most important to the carer. This is then used to help consider what is most important value in specific situations, e.g. connection. |
|  | Self-Care Tools | An exploration of a range of self-care tools, building on the self-care battery introduced at the start of the course. Not every tool will be right for everyone. |
| **Week 6 - Summing up - Drawing the Course to a Close** | Tony Husband Cartoon | Using a Tony Husband cartoon, representing a person living with dementia and a carer, to start discussion regarding some ways communication can be impaired and how each of the people are feeling |
|  | ‘Cartoon’ - draw an example of a conversation that did not go quite as carer had hoped it would. | Care partners are asked (if they feel comfortable with this) to think of a scenario with a person they know who is living with dementia where there were barriers to communication/listening and how the conversation looked, then draw this. |
|  | Summing up with Stories of Change | Celebrate peoples’ stories at this point towards the end of the course. These stories of change do not have to be massive; they can be that somebody has been able to recognise when they are getting frustrated and take themselves away from the situation and do the breathing exercise! |
